# Management of late onset urea cycle disorders—a remaining challenge for the intensivist?

**DOI:** 10.1186/s13613-020-00797-y

**Published:** 2021-01-06

**Authors:** S. Redant, A. Empain, A. Mugisha, P. Kamgang, R. Attou, P. M. Honoré, D. De Bels

**Affiliations:** 1 Department of Intensive Care, Université Libre de Bruxelles (ULB), CHU Brugmann–Brugmann University Hospital, 4, Place Arthur Van Gehuchten, 1020 Brussels, Belgium; 2 Department of Metabolic Diseases, Hôpital universitaire des enfants reine Fabiola, Université Libre de Bruxelles (ULB), Brussels, Belgium; 3 Department of Internal Medicine, Brugmann University Hospital, Université Libre de Bruxelles (ULB), Brussels, Belgium

**Keywords:** Urea cycle disorders, Hyperammonemia, Dialysis, Scavenging therapy

## Abstract

**Background:**

Hyperammonemia caused by a disorder of the urea cycle is a rare cause of metabolic encephalopathy that may be underdiagnosed by the adult intensivists because of its rarity. Urea cycle disorders are autosomal recessive diseases except for ornithine transcarbamylase deficiency (OTCD) that is X-linked. Optimal treatment is crucial to improve prognosis.

Main body

We systematically reviewed cases reported in the literature on hyperammonemia in adulthood. We used the US National Library of Medicine Pubmed search engine since 2009. The two main causes are ornithine transcarbamylase deficiency followed by type II citrullinemia. Diagnosis by the intensivist remains very challenging therefore delaying treatment and putting patients at risk of fatal cerebral edema. Treatment consists in adapted nutrition, scavenging agents and dialysis. As adults are more susceptible to hyperammonemia, emergent hemodialysis is mandatory before referral to a reference center if ammonia levels are above 200 µmol/l as the risk of cerebral edema is then above 55%. Definitive therapy in urea cycle abnormalities is liver transplantation.

**Conclusion:**

Awareness of urea cycle disorders in adults intensive care units can optimize early management and accordingly dramatically improve prognosis. By preventing hyperammonemia to induce brain edema and herniation leading to death.

## Background

Hyperammonemia caused by a disorder of the urea cycle is a rare cause of metabolic encephalopathy that may be underdiagnosed by the adult intensivists because of its rarity. An acute ammonia elevation, if left untreated, leads to brain swelling, structural damage and death [1]. Ammonia diffuses freely across the blood–brain barrier and is converted with alanine to glutamine by glutamine synthase. Glutamine is the main intracellular osmole of the brain. Its accumulation causes the swelling of astrocytes during hyperammonemia (Alzheimer type II astrocyte) [2].

**Fig. 1:**
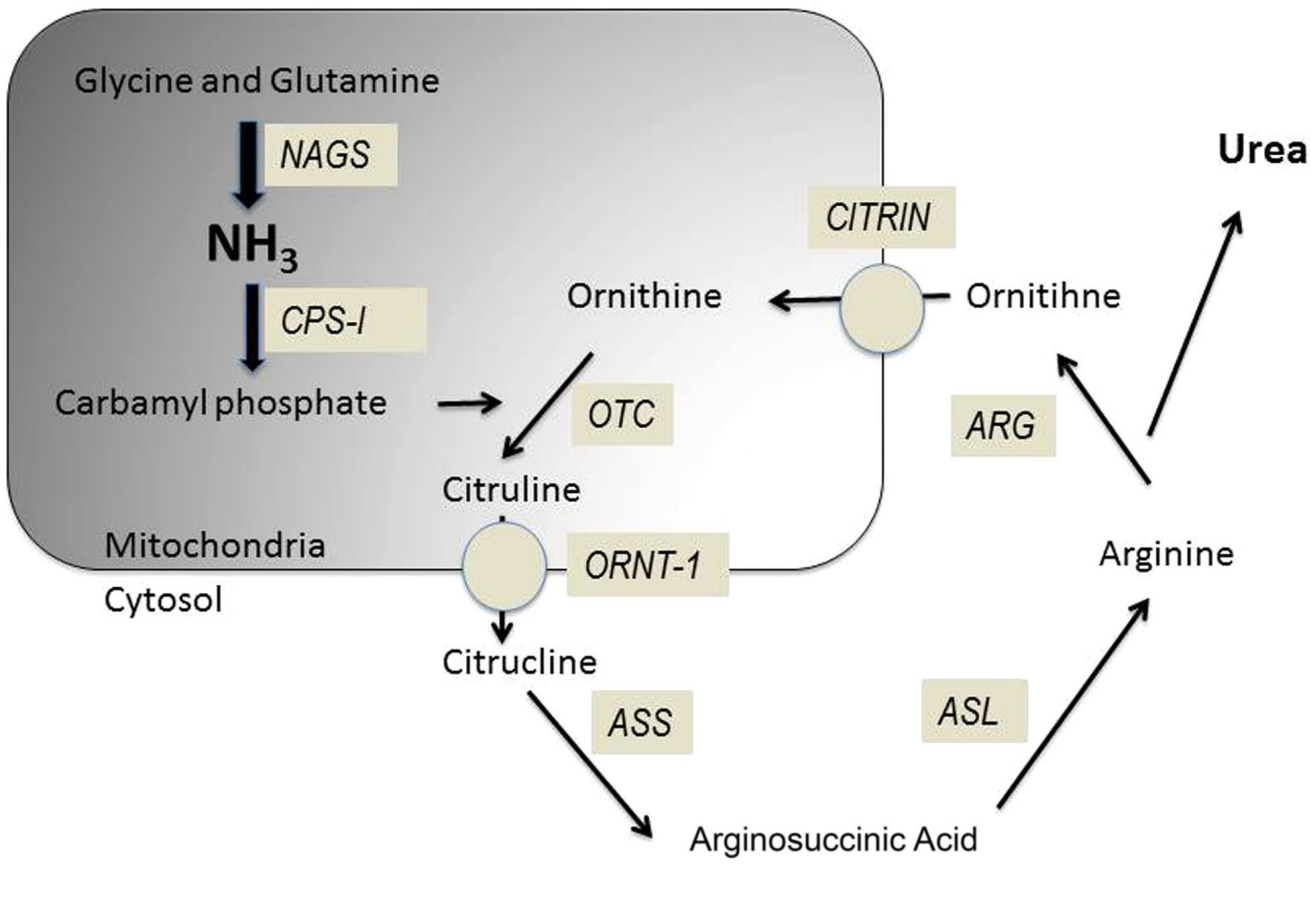
Urea cycle disorders: NAGS: *N*-acetyl glutamate synthetase, CPS1: Carbamyl phosphate synthetase 1, OTC: Ornithine transcarbamylase, ORNT1 mitochondrial ornithine transporter 1, CITRIN: Citrullinemia type II (mitochondrial aspartate/glutamate) carrier, ASS: Arginosuccinic acid synthetase 1, ASL: Arginosuccinic acid lyase

Urea cycle disorders (UCD, Fig. 1) are autosomal recessive diseases except for ornithine transcarbamylase deficiency (OTCD) that is X-linked [3]. The annual incidence in the United States is 1: 35,000 births which represents 113 new cases per year [4]. The UCDs comprise 8 abnormalities listed in Table 1 altering cofactors, enzymes or transporters [3]. The usual presentation of urea cycles disorders is neonates with extremely high levels of ammonia and coma. This model constitutes the majority of the reported cases in the literature. However, some cases are reported with onset in adulthood. A partial or moderate deficiency in a urea cycle enzyme allows patients to evolve without incident until adulthood [5]. These patients avoid proteins in their diet, some to the point of becoming vegetarian. The onset of symptoms coincides with a precipitating factor such as excess protein intake, infection, trauma, surgery, deliverance [6] or medications as valproate or glucocorticoids [7]. The authors reviewed systematically the clinical cases in the literature in order to obtain information on presentation and management of patients with an UCD in adulthood. We wish to focus our review on the different therapeutic possibilities with which the intensivist must be familiar.

**Table 1:** The various deficiencies of urea cycle disorders adapted from Waisbren [3]

Cofactor
*N*-Acetylglutamate synthase deficiency (NAGSD)
Enzymes
Carbamyl phosphate synthetase 1 deficiency (CPS1D)
Ornithine transcarbamylase deficiency (OTCD)
Argininosuccinate synthetase deficiency (ASSD) (citrullinemia)
Argininosuccinate lyase deficiency (ASLD) (Argininosuccinic aciduria)
Arginase deficiency (ARGD, argininemia)
Transporter
Hyperornithinemia, hyperammonemia, homocitrullinuria (HHH) syndrome (or mitochondrial ornithine transporter 1 deficiency (ORNT1D)
Citrullinemia type II (mitochondrial aspartate/glutamate carrier deficiency (CITRIN)

## Main text

### Pathophysiology

During metabolic stress induced by infection, childbirth or surgery, a catabolic phase leads to the metabolism of a large amount of proteins. This excess intake will exceed the capacity of the urea cycle, especially if it has an enzymatic deficit. A significant production of ammonia then follows. Ammonia passes into the circulation and crosses the blood–brain barrier. The ammonia will exert a direct toxic effect on the neurotransmission responsible for part of the neurological symptomatology. In addition, the astrocytic glutamine synthetase will convert ammonia and glutamate into glutamine, which in turn acts as an osmolyte and will increase cerebral volume [8]. Intra cranial hypertension appears inducing coma, cerebral engagement and death of the patient.

A particular form is the form induced by Valproate. Valproate causes hyperammonemia by blocking carbamoyl phosphate synthetase, which is an enzyme at the beginning of the urea cycle (Fig. 1). The second mechanism is the inhibition of carnitine transport in the mitochondria, which causes a shift toward protein degradation responsible for hyperammonemia [14]. In the case of an urea cycle disorder, the blocking of carbamoyl synthetase and the increase of protein degradation favor or exacerbate the development of a hyperammonemic crisis.

### Literature

**Fig. 2:**
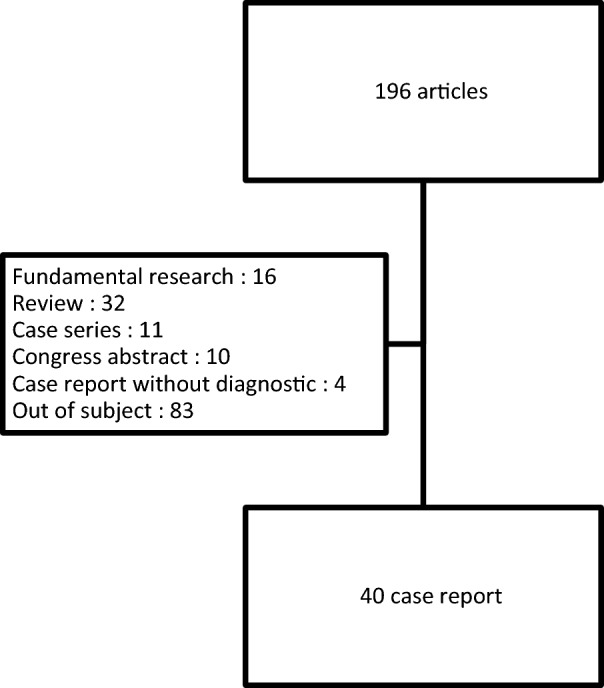
Literature flow chart of analyzed case reports

We conducted a systematic review of the case-reports described in the literature [1, 6, 7, 9–34]. We used the US National Library of Medicine Pubmed search engine with the following key words: "adult-onset”, “Late-onset”, and “urea cycle disorder”, “inborn urea metabolism disorder”. We deliberately excluded cases-series publications, reviews, fundamental research articles and the case reports where the final diagnosis was not confirmed (Fig. 2).

**Fig. 3:**
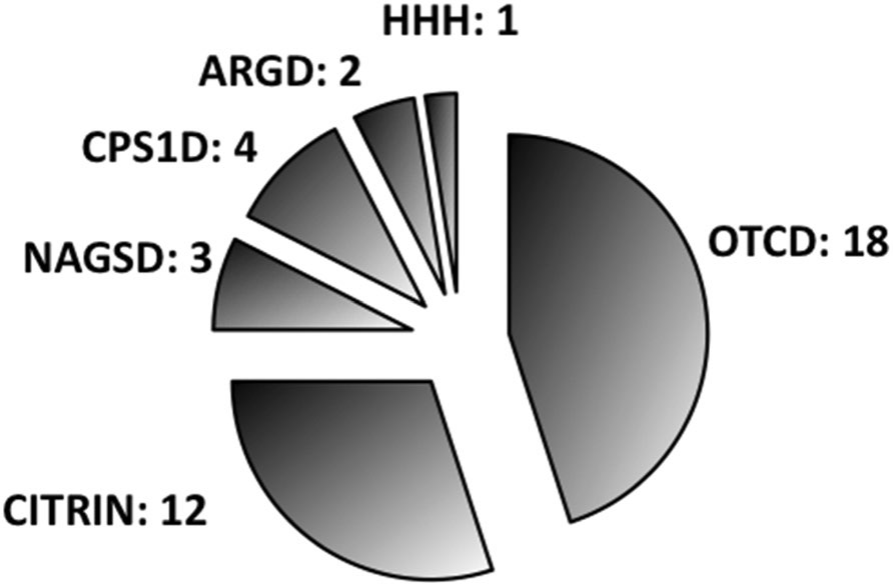
Distribution of different anomalies in the urea cycle. CPS1D: Carbamyl phosphate synthetase 1 deficiency, OTCD: Ornithine transcarbamylase deficiency, HHH: Hyperornithinemia, hyperammonemia, homocitrullinuria (HHH) syndrome; ORNT1D mitochondrial ornithine transporter 1 deficiency, CITRIN: Citrullinemia type II (mitochondrial aspartate/glutamate carrier deficiency

We found 40 adult case-reports meeting our selection criteria (Table 2). The average ammonia level measured at admission is 280 (162–480) µmol/l with a mean peak of 300 (228–541) µmol/l. Symptoms are listed in Table 3, the most common being confusion. Cerebral edema was highlighted in only 13 cases. The most frequently found favoring factors were infection and type of nutrition. Other factors are listed in Table 4. Eight patients spontaneously adopted protein-free diets before diagnosis was made. Nineteen patients were treated with benzoate, 2 with phenylbutyrate and 8 with the combination of both. Fifteen patients received l-arginine, 1 patient received l-carnitine and 6 received the combination of both. Four patients received citrulline. Dialysis was used in 14 patients and hypothermia in only one patient (Table 5). The most frequently found anomaly was ornithine transcarbamylase (OTC) deficiency with the same male / female ratio followed by citrullinemia type II (Fig. 3). Height patients died. Ninety-four publications concerned other metabolic errors than the urea cycle.

**Table 2:** Adult case-reports of late onset urea cycle disorders

N°	Ref	Sex	Age	Acute symptoms	Maximal ammonia	Brain edema	Defective enzyme	Acute treatment	Dialysis	Outcome
1	[34]	M	48	Coma, headache, S, N, V	497	Yes	ORNT1	B	Yes	F
2	[33]	F	19	N, V	ND	Yes	OTC	Other	No	D
3	[32]	F	52	BA, headache, S, N, V	330	No	NAGS	B	No	F
4	[31]	M	69	N, V	293	No	OTC	Other	Yes	F
5	[30]	M	66	Coma, BA, S	494	Yes	OTC	Other	Yes	F
6	[29]	M	60	Coma	158	Yes	ARG	B	No	F
7	[7]	M	67	S	ND	Yes	OTC	Other	No	F
8	[28]	F	52	Coma	684	No	OTC	Other	No	F
9	[1]	F	39	Coma, N, V	288	No	OTC	B	Yes	F
10	[27]	K	62	Behavior, S	154	No	CITRIN	Other	No	F
11	[26]	F	73	Coma, Behavior	147	No	CITRIN	Other	Yes	F
12	[6]	F	59	Coma	280	No	NAGS	B	No	F
13	[25]	M	31	BA	598	No	CITRIN	Other	No	F
14	[24]	F	48	BA, headache	500	No	CITRIN	B + LT	No	F
15	[23]	M	38	BA, headache, N, V	434	No	NAGS	PB	No	F
16	[22]	M	28	Coma	683	Yes	CPS1	B + PB	No	D
17	[21]	M	49	Coma	254	No	OTC	B	Yes	D
18	[20]	F	35	BA, headache	224	No	CPS1	B + PB	No	F
19	[19]	M	59	Coma, S, N, V	228	No	OTC	B	Yes	F
20	[18]	F	23	Headache, V	477	No	OTC	B + PB	No	F
21	[17]	M	47	S, V	541	Yes	OTC	−	Yes	F
22	[16]	M	49	S, N	157	No	CPS1	B + LT	No	F
23	[15]	M	45	S	628	Yes	OTC	−	No	D
24	[14]	F	31	Other	179	No	OTC	PB	No	F
25	[13]	M	17	S, N, V, Coma	787	Yes	OTC	B	Yes	D
26	[12]	M	63	S, coma	1447	Yes	OTC	B + PB	Yes	D
27	[11]	F	28	Coma	281	No	OTC	B + PB	Yes	F
28	[10]	F	60	S, BA, Coma, N, V	256	No	CPS1	B + PB	Yes	D
29	[9]	F	25	N, V	150	No	CITRIN	PB	No	F
30	[53]	M	36	BA	696	No	OTC	B + PB	No	F
31	[54]	F	18	S,N,V	23	No	ARG	Other	No	F
32	[55]	F	40	BA, Coma	300	No	CITRIN	Other	No	F
33	[56]	M	40	BA, Coma	390	No	OTC	B	Yes	F
34	[57]	F	21	Coma, N, V	510	No	OTC	B	No	F
35	[58]	M	34	BA, Coma	2210	Yes	CITRIN	Other	No	D
36	[59]	F	21	S, BA, Coma	698	Yes	CITRIN	LT	No	F
37	[60]	M	31	BA	396	Yes	CITRIN	Other	No	D
38	[61]	M	31	Other	263	No	CITRIN	LT	No	F
39	[62]	M	41	BA	483	No	CITRIN	B	Yes	F
40	[63]	F	40	S, BA	234	No	CIRTIN	Other	No	F

M: male; F: female; S: seizure; N: nausea; V: vomiting, BA: behavioral abnormalities; ORNT1: ornithine transporter 1; OTC: ornithine transcarbamylase; NAGS: N-Acetylglutamate synthase; ARG: arginase; CITRIN: aspartate/glutamate carrier; CPSI: Carbamyl phosphate synthetase 1; B: benzoate; PB: phenylbutyrate; LT: liver transplantation; D: died; F: favorable

**Table 3:** Frequency of different symptoms presented by patients

Symptoms	Frequency	%
Confusion	18	45
Vomiting	17	42
Convulsions	14	35
Coma	17	42
Behavioral disorders	14	35
Nausea	15	37
Headaches	6	15
Loss of consciousness	8	20

**Table 4:** Frequency of factors favoring decompensation

Circumstances	Patients (*n*)	%
Infection	5	12
Parenteral nutrition	2	5
Hyper-protein diet	2	5
Valproic acid	2	5
Post-partum	2	5
Post-operative	1	2.5
Myocardial infarction	1	2.5
Gastrointestinal bleeding	1	2.5
Unknown	23	57

**Table 5:** Different treatment type frequency

Treatment	Patients (*n*)	%
Low protein oral nutrition	28	73
Benzoate	27	46
L-Arginine	21	50
Dialysis	14	34
Phenylbutyrate	10	19
L-Carnitine	7	27
Citruline	4	15
Low protein parenteral nutrition	7	15
Hepatic transplantation	4	7

### Diagnosis

**Fig. 4:**
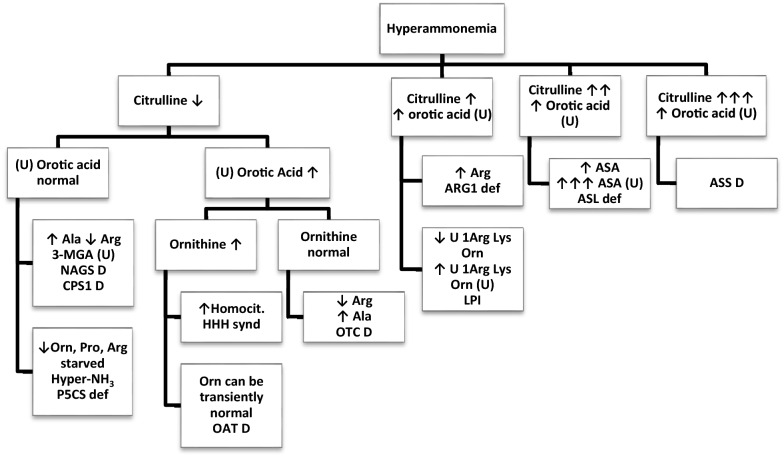
diagnostic algorythm for late onset hyperammonemia. In case of hyperammonemia, the levels of citrulin and urinary orotic acid makes it possible to direct towards a specific analysis group leading to the final diagnosis. Unless indicated, with (U) to indicate urine. Ala: alanine, Arg: arginine, ARG1: arginase 1, 3-MGA: 3-methylglutaconic acid, NAGS: N-acetylglutamate synthase, CPS: carbamoylphosphate synthetase, Orn: ornithine, Pro: proline, P5CS: pyrroline-5-carboxyl synthetase, HHH: hyperornithinemia-hyperammonemia-homocitrullinuria, OAT: ornithine aminotransferase, OTC: ornithine transcarbamylase, Lys: lysine, LPI: lysinurix protein intolerance, ASA: arginosuccinic acid, ASS: arginosuccinate synthetase

The diagnosis is made difficult by the non-specific nature of symptoms. In 42 percent of cases, patients do not receive a scavenging treatment. Twelve patients received neither arginine nor l-carnitine. In 5 cases, the situation was considered out of date, which led to a lack of treatment. The time to initiate treatment was not specified. Despite high levels of ammonia (27 patients above 250 µmol/l), dialysis was only offered in 14 patients. In the height patients who died, only 4 were dialyzed. Bernal et al. showed that an ammonia level > 100 µmol/ml predicted the occurrence of severe encephalopathy with 70% accuracy. They also observed that 55% of patients with ammonia levels > 200 µmol/l had Intracranial hypertension [35]. In hyperammonemia associated with urea cycle disorders, treatment with hemodialysis can reverse encephalopathy and prevent brain edema and death [36]. The diagnosis of abnormalities of the urea cycle requires a determination of organic and urinary amino acids, acylcarnitines and follows an algorithm shown in Fig. 4 [37].

### Clinical presentation

A slower rise in ammonia increases brain levels of tryptophan, a precursor of serotonin. Increased serotonin production may contribute to behavioral abnormalities, migraine, headaches, and changes in cerebral blood flow [2]. It is strongly recommended to consider an UCD at any age in the presence of an acute or intermittent neurological deterioration or a psychiatric pathology, an acute hepatic failure or intoxication. The trigger is a catabolic state or a high protein load [37].

### Acute management in intensive care

**Fig. 5:**
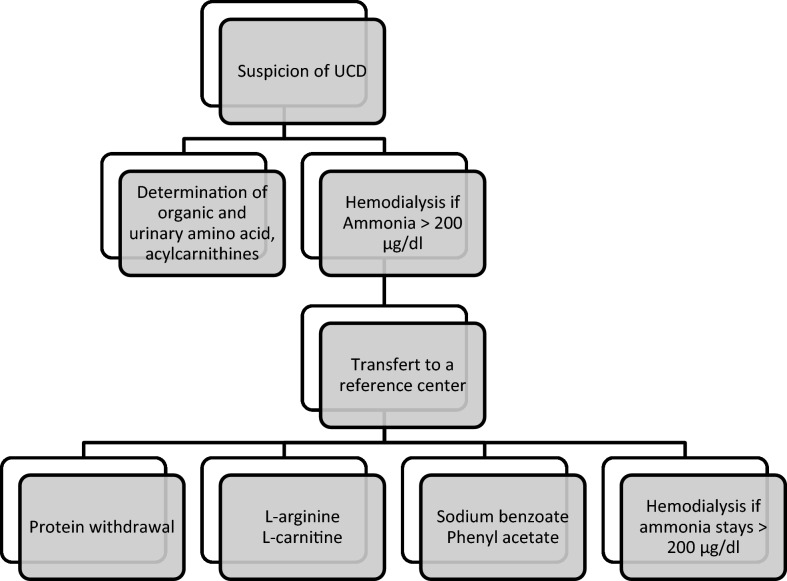
Management of UCD

**Fig. 6:**
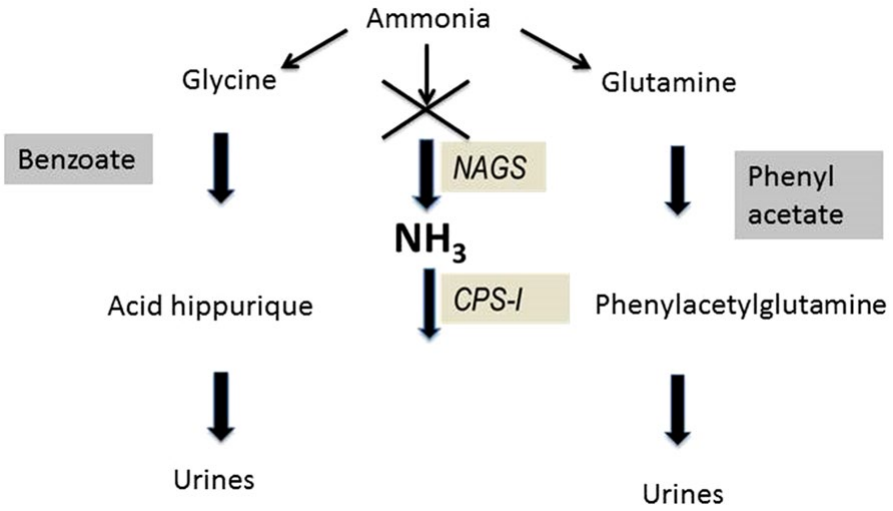
Alternative pathways to reduce ammonia production and accelerate elimination. Ammonia is diverted to the glycine and hippuric acid pathway by benzoate, and to the glutamine and phenylacetylglutamine pathway allowing elimination in the urine without passing through the urea cycle

The treatment is clearly defined in the literature (Fig. 5) and can be started regardless of the type of enzyme deficiency underlying the urea cycle [38, 39], the goal of treatment being to reduce ammonia production and accelerate elimination via alternative pathways (Fig. 6).

#### Nutritional management

Acute management of an enzyme deficiency in the urea cycle includes stopping any exogenous protein supply during 48 h. Proteins must be re-introduced after 48 h to avoid endogenous protein catabolism. Energetic intake should be provided by infusion of dextrose 10–30% and a 20% fat emulsion (Intralipid®) to provide supranormal caloric intake to avoid catabolism with a transition as soon as possible to an oral diet with anti-emetics if necessary. The proteins are then reintroduced in a second time. Supplementation with L-Arginine or Citrulline is recommended to promote an alternative pathway of metabolism [5].

#### l-arginine and l-carnitine

Patients with defective urea synthesis could have a low arginine level which induces in these patients increased proteolysis as the degradation/synthesis cycle is interrupted. The administration of arginine in those patients limits proteolysis and thus promotes the reduction of urea [40]. l-Carnitine (LC) provides cerebral protection in case of hyperammonemia. LC crosses the blood brain barrier and causes alanine to drop by restoring mitochondrial respiration by improving pyruvate oxidation, Krebs cycling, and flux through glutamate dehydrogenase. It is via this effect on glutamate dehydrogenase that it is also explained a drop in the level of ammonia blood post administration of LC [41].

The administration of systematic arginine is subject to debate. In our review one patient out of 29 (3.4%) had an arginase deficiency which lead to an increase in blood arginine level. Administering arginine under these conditions would increase the already high level of arginine. However, to our knowledge, there are no pathological repercussions of a high level of arginine.

#### Scavenging agents

Sodium benzoate, by the acetylation of glycine on hippuric acid, can extract one mole of nitrogen for each mole of benzoate administered. Sodium phenylacetate is conjugated with glutamine to form phenylacetylglutamine which is eliminated by the kidney (Fig. 6). Glutamine contains 2 nitrogen atoms, so each mole of sodium phenylacetate removes 2 mol of nitrogen. These conversions take place in the liver and kidney [42]. The main side effects of these drugs administered in IV are nausea, vomiting and hypokalemia [43]. Brusilow et al. reported the efficacy of sodium benzoate combined phenylacetate and arginine in patients with UCD in hyperammonemic crisis [40]. Enns et al. showed a 98% survival rate in pediatric patients older than 30 days who presented with an hyperammonemic attack. This value reached 99% for children over 12 years old [44].

#### *N*-carbamoyl-l-glutamic acid

*N*-Carbamoyl-l-glutamic acid (NCG or Carbaglu®) is a structural analogue of *N*-acetyl glutamate (NAG) that restores urea cycle function in inherited NAGS and CPS1 deficiency [45]. It works by replacing NAG which is an essential activator of CPS1 [46]. NCG has also shown its effectiveness in OTC deficiency. On one hand, since the activity of the OTC is decreased, it increases the pool of carbamyl phosphate available for this enzyme. On the other hand, it would increase the stability of the enzyme by promoting binding with carbamyl phosphate [47].

NCG is potentially useful in all other enzymatic deficiency urea cycle. It has been experimentally demonstrated that fasting and protein-free dieting, which are the cornerstones of UCD hyperammonemia management, lead to a decrease in NAG activity following a down-regulation mechanism and increased the activity of other downstream enzymes [48]. NCG is used to stimulate the residual activity of enzymes in the urea cycle. In addition, it acts as an activator of the urea cycle and has been proposed as a potential treatment for hyperammonemia secondary to organic acidemia, hepatic encephalopathy and even valproic acid associated hyperammonemia [46, 47].

#### Dialysis

In 1979, Donn et al. showed the effectiveness of hemodialysis in the clearance of ammonia in a patient with OTC deficiency. The measured clearance was 12,600 μg/h. This value was much higher than the value obtained by peritoneal dialysis or transfusion exchange [49]. Ammoncia has a clearance as important as urea and is therefore easily dialyzable.

Enns et al. reported that only 12% of hyperammonemia episodes were treated with renal replacement therapy. Most of them were treated with a combination of phenylacetate and benzoate treatment [44]. Batshaw et al. suggested in a review of 20 years of use of alternative pathway therapy that in hyperammonemia comas (ammonia > 250 μmol/l), benzoate is insufficient even when combined with phenylacetate. Dialysis should be considered while maintaining benzoate as both therapies could potentially be synergistic [42]. The current recommendations are to consider hemodialysis in adults from a cut-off at 200 µg/dl. Hemodialysis is the first-choice treatment because it is available everywhere and must be started before being transferred to a center specialized in metabolic pathologies where other medical treatments are available [37]. Given the rebound effect, it is advisable to continue with a continuous veno-venous hemodialysis (CVVHD). Some authors argue to replace HD with high-flow CVVHD followed by conventional dose CVVHD when the ammonia is less than 150 µmol/l [50].

#### Liver transplantation

Liver transplantation is the definitive cure for urea cycle abnormalities. Transplantation is considered only in patients with recurrent hyperammonemia or resistant to medical treatment [51]. Moriorka et al. reported 51 cases of patients with urea cycle disorder who beneficiated from liver transplantation with a good result and a good quality of life. The need of dietary restriction and scavenging agents were totally eliminated after transplantation [52].

### Chronic management outside the intensive care

Chronic treatment is initiated and followed by the specialists in metabolic disease. It consists of a strict diet, supplementation with l-Arginine and l-carnitine and possibly a scavenging therapy adapted to the case [37].

## Conclusion

Hyperammonemia associated with a urea cycle abnormality is a therapeutic emergency to prevent brain herniation and death. Having a high suspicion is important. The current recommendations are to consider hemodialysis in adults from a cut-off at 200 µg/dl. Hemodialysis is the first-choice treatment before transferring the patient to a tertiary reference center where specific treatment options are available. The final diagnosis can be made after resolution of the hyperammonemia crisis and needs a close collaboration with the metabolic disorder specialist.

## Data Availability

Not applicable.

## Abbreviations

UCD: Urea cycle disorders
OTCD: Ornithine transcarbamylase deficiency
LC: L-carnitine
NCG: *N*-Carbamoyl-l-glutamic acid
OTC: Ornithine transcarbamylase
ARG: Arginase
NAGS: *N*-Acetylglutamate synthase
ASS: Argininosuccinate synthetaseS)
CPS1: Carbamyl phosphate synthetase 1
ASL: Argininosuccinate lyase
ORNT1: Mitochondrial ornithine transporter 1
CITRIN: Mitochondrial aspartate/glutamate carrier
